# A Modular Organization of the Human Intestinal Mucosal Microbiota and Its Association with Inflammatory Bowel Disease

**DOI:** 10.1371/journal.pone.0080702

**Published:** 2013-11-19

**Authors:** Maomeng Tong, Xiaoxiao Li, Laura Wegener Parfrey, Bennett Roth, Andrew Ippoliti, Bo Wei, James Borneman, Dermot P. B. McGovern, Daniel N. Frank, Ellen Li, Steve Horvath, Rob Knight, Jonathan Braun

**Affiliations:** 1 Department of Molecular and Medical Pharmacology, David Geffen School of Medicine, University of California Los Angeles, Los Angeles, California, United States of America; 2 Cedars-Sinai F. Widjaja Inflammatory Bowel and Immunobiology Research Institute, Los Angeles, California, United States of America; 3 Department of Chemistry & Biochemistry, University of Colorado, Boulder, Colorado, United States of America; 4 Department of Medicine, Division of Digestive Disease, David Geffen School of Medicine, University of California Los Angeles, Los Angeles, California, United States of America; 5 Department of Pathology and Laboratory Medicine, David Geffen School of Medicine, University of California Los Angeles, Los Angeles, California, United States of America; 6 Department of Plant Pathology and Microbiology, University of California Riverside, Riverside, California, United States of America; 7 Division of Infectious Diseases, University of Colorado, School of Medicine, Aurora, Colorado, United States of America; 8 Union Council, Denver Microbiome Research Consortium (MiRC), University of Colorado, School of Medicine, Aurora, Colorado, United States of America; 9 Department of Medicine, Stony Brook University, Stony Brook, New York, United States of America; 10 Department of Human Genetics and Biostatistics, David Geffen School of Medicine, University of California Los Angeles, Los Angeles, California, United States of America; 11 Howard Hughes Medical Institute, University of Colorado, Boulder, Colorado, United States of America;; Massachusetts General Hospital, United States of America

## Abstract

Abnormalities of the intestinal microbiota are implicated in the pathogenesis of Crohn's disease (CD) and ulcerative colitis (UC), two spectra of inflammatory bowel disease (IBD). However, the high complexity and low inter-individual overlap of intestinal microbial composition are formidable barriers to identifying microbial taxa representing this dysbiosis. These difficulties might be overcome by an ecologic analytic strategy to identify modules of interacting bacteria (rather than individual bacteria) as quantitative reproducible features of microbial composition in normal and IBD mucosa. We sequenced 16S ribosomal RNA genes from 179 endoscopic lavage samples from different intestinal regions in 64 subjects (32 controls, 16 CD and 16 UC patients in clinical remission). CD and UC patients showed a reduction in phylogenetic diversity and shifts in microbial composition, comparable to previous studies using conventional mucosal biopsies. Analysis of weighted co-occurrence network revealed 5 microbial modules. These modules were unprecedented, as they were detectable in all individuals, and their composition and abundance was recapitulated in an independent, biopsy-based mucosal dataset 2 modules were associated with healthy, CD, or UC disease states. Imputed metagenome analysis indicated that these modules displayed distinct metabolic functionality, specifically the enrichment of oxidative response and glycan metabolism pathways relevant to host-pathogen interaction in the disease-associated modules. The highly preserved microbial modules accurately classified IBD status of individual patients during disease quiescence, suggesting that microbial dysbiosis in IBD may be an underlying disorder independent of disease activity. Microbial modules thus provide an integrative view of microbial ecology relevant to IBD.

## Introduction

Inflammatory bowel disease (IBD), a spectrum of chronic, relapsing inflammatory intestinal diseases, results from the interaction of environmental factors, including intestinal microbiota, with host immune mechanisms in genetically susceptible individuals [1,2]. Human and animal studies demonstrate the involvement of intestinal microbiota in the onset or perpetuation of inflammation, and intensive efforts have search for individual bacterial species and specific bacterial products in the pathogenesis of IBD [3-5]. However, rather than revealing a single agent responsible for disease, these studies have uncovered a variety of bacterial taxa and products that can either promote or attenuate the inflammatory disease state. Moreover, the relevant microbiota differ in accord with the genetic susceptibility traits of the host [6-9]. These insights have shifted the concept of microbial pathogenesis in IBD away from specific pathogens and towards ecologic, community-level change [10], and raised concomitant challenges of establishing coherent concepts and analytic strategies to identify microbiota relevant to disease risk or disease activity in individual IBD patients. 

In recent years, the phylogenetic and functional characterizations of the human enteric microbiota in IBD have been elucidated with the help of second-generation sequencing platforms. One striking feature of human intestinal microbiome is its great inter-individual phylotypic variation [11-13]. This variability has complicated the association of microbial phylogenetic composition with disease, in that it is challenging to determine if the absence of a given phylotype in a healthy or disease subject is due to the pathogenic physiology or simply temporal or inter-individual stochastic fluctuations. Although a “core microbiome” at the gene level is identifiable [14], the core feature at the organismal lineage level, which resolves functionally redundant phylotypes into distinct communities, has not yet been defined. In the context of IBD microbial pathogenesis, this has prompted the current concept that an individual’s distinct microbial composition (shaped by host genetics, founder effects, and diet) may create a disease-susceptible ecology prone to blooms of pathobionts (and/or busts of protective taxa) when stressed by environmental, metabolic, or viral disturbances [15]. However, due to the limitations of current microbial analysis, reproducible microbial features established for human IBD are quite limited: reduced alpha diversity, and a small number of elevated or reduced taxons detectable at the level of patient categories but only sporadic at the level of individual patients. Accordingly, these findings are sufficient neither to test the current pathogenesis concept, nor to provide a strategy to classify and monitor individual patients for disease-associated microbial taxa. 

To validate this concept and allow clinical translation, we must move beyond existing studies of taxon and/or gene composition to instead quantify relevant features of the microbial community at the ecological level. Extensive inter-species interactions exist in the highly complex intestinal microbial ecosystem [16,17]. Investigating the hundreds of thousands of possible pairwise inter-species interactions in a defined system is not feasible [18], especially because few known intestinal microbes are cultivable. 16S rRNA gene profiling allows us infer inter-species correlations from relative abundance profiles. Several benchmarking studies have documented microbial co-occurrence in different environments [19-22], but the role of inter-species interactions during the pathogenesis of chronic disease remains largely unexplored. Here we adopted a methodology for phylogenetic network analysis to search for such interactions, suggesting that the human mucosal surface bacterial community is organized into 5 highly preserved modules. Two of these modules are reciprocally associated with inflammatory bowel disease. 

## Materials and Methods

### Patient cohorts and lavage sample collection

A previously assembled patient cohort of 64 subjects [23] was examined (Table S1) in accord with human subject protocols approved by the institutional review boards of University of California Los Angeles and Cedars Sinai Medical Center. This included written informed consent of each subject to participate in the study, performed per protocol approved by the institutional review boards, which serves as the institutional ethics committee for human subjects research. All enrolled subjects were prepared for colonoscopy by taking Golytely® the day before the procedure. The mucosal lavage samples representing the mucosal luminal interface were collected from different intestinal regions as described previously [23]. All the lavage samples in this cohort were collected from non-involved intestinal regions, which excluded the potential influence of active inflammation on the mucosal microbiota as much as possible. Subjects metadata, including diagnosis, gender, age, and colon regions sampled, were recorded. The influence of medication on the microbiome was not evaluated, due to the unavailability of data. 

### 16S rRNA Gene Sequencing and Microbial Composition Analysis

After collection, the sample was centrifuged at 3,500g for 15 minutes to separate the microbiota from the soluble fraction. Genomic DNA was extracted as described in Costello et al. [24]. The hyper-variable region 4 of the 16S ribosomal RNA gene was then amplified and sequenced on an Illumina HiSeq 2000 as described in Caporaso et al. [25]. The sequence data is deposited in European Bioinformatics Institute [EMBL: ERP001780]. The median read length of sequences that passed quality filtering is 90 bp and the average read length is 88 bp with a filtering threshold of 75bp. For quality control, all the singletons were removed, and samples with fewer than 3,000 reads were excluded from the following analyses. The 97% OTUs were picked against the Greengenes reference database (February 4th, 2011) first, then reads that did not match a Greengenes sequence at 97% or greater sequence identity were clustered *de novo* using uclust [26]. Taxonomy of each OTU was assigned by blasting the representative sequence against Greengenes reference database [27] (http://greengenes.lbl.gov/cgi-bin/nph-index.cgi). These steps were performed using Quantitative Insights Into Microbial Ecology (QIIME) v1.4.0 [28]. 

Alpha rarefaction was performed using the Phylogenetic Diversity index. Ten sampling repetitions were performed at each sampling depth ranging from 10 to 3,000 reads. The comparison of alpha diversity between two groups at certain sampling depths was performed using a two-sided Student *t* test. Significance was defined as a P value of less than 0.05. Beta diversity was estimated by computing unweighted UniFrac distances between samples using QIIME. Principal coordinates analysis (PCoA) was applied to reduce the dimensionality of the resulting distance matrix. 

### Construction of microbial co-occurrence network

We first defined a co-occurrence similarity measure which was used to define the network. Assume that the vector x_*i*_ specifies the abundance of the *i*-th genus across the samples, the pair-wise Sparse Correlations for Compositional data (SparCC) *ρ*_*ij*_ was inferred from the abundance profile of each genus x_*i*_ and x_*j*_ as the measurement of co-occurrence relationship. A signed weighted adjacency matrix (network) was defined by raising *ρ*_*ij*_ to a power *a*_*ij*_ = (0.5 + 0.5*ρ*_*ij*_) ^ β, with β = 4 [29]. The power is a soft threshold that preserves the continuous nature of the underlying co-occurrence information. The relatively low power of 4 (chosen with the scale free topology criterion) likely reflected the fact that the network was comprised of relatively few nodes (263 genera). Once the network was constructed, modules were then defined as branches of a hierarchical clustering tree based on the topological overlap measure, because it is a highly robust measure of network interconnectedness. The modules were detected after applying the dynamic tree cut method [30]. These network modules (clusters) were interpreted as functional microbial communities (FMCs). To summarize the profiles of co-occurrence modules, we calculated the eigengenus, which provides a mathematically optimal way of summarizing the co-occurrence patterns of all genera belonging to each module. To identify modules (FMCs) that were correlated with clinical traits, we used correlation tests to relate each eigengenus to the clinical traits. These steps were performed using WGCNA package (version 1.13) in R (version 2.13.1) [31]. R tutorials explaining the analysis steps can be found on the webpage: http://www.genetics.ucla.edu/labs/horvath/CoexpressionNetwork/Rpackages/WGCNA/Tutorials/. 

### Module preservation analysis

Meta-analysis was performed with two mucosal microbial datasets: the previously published “Frank” dataset [32], and the “Tong” dataset presented in this paper (using the same biospecimens described in a recently reported patient cohort [23]). Prior to meta-analysis, the taxonomy of each OTU in Frank dataset was re-assigned by blasting the representative sequence against Greengenes reference database. The common phylotypes at genus level that were present in both dataset were then identified. After this filtering step, 1,196,466 out of 1,236,641 reads (96.8%), or 129 out of 263 genera in Tong dataset, and 13,165 out of 15,172 reads (86.8%), or 129 out of 263 genera in Frank dataset were included in the following analysis. To determine whether a FMC found in the reference dataset was also present in the test dataset, we used a powerful module preservation statistic implemented in the R software function modulePreservation [33]. For each module, the aggregate measure of module preservation was termed the preservation Z-summary statistic. The higher the value of the Z-summary statistic is for a given module, the stronger the evidence that the module is preserved in the test dataset. Comprehensive simulation studies led to the following thresholds: a module shows no evidence of preservation if its Z-summary statistic is smaller than 2; a Z-summary statistic larger than 5 (or 10) indicates moderate (strong) module preservation. 

### Imputation of microbial gene content and metagenomes of FMCs

The OTU table of the 5 FMCs in Tong dataset was generated with 1 count for each 97% OTU in a given FMC. The gene content of 1,119 KEGG reference genomes was used to infer the approximate gene content of the detected 97% OTUs in our dataset using Phylogenetic Investigation of Communities by Reconstruction of Unobserved States (PICRUSt) (v0.1). The functional traits copy numbers of the reference genomes represented in the format of KEGG KO functions can be downloaded from the PICRUSt website (http://picrust.github.com). To predict the functional traits of non-sequenced microbial genomes (i.e. 97% OTUs) in Tong dataset, a phylogenetic tree of 97% OTUs in Greengenes database was constructed using 16S marker gene. The tree has tips representing both sequenced referenced genomes and non-sequenced genomes. Then the ancestral state reconstruction (ASR) was run for this tree to make predictions for each KO functions for every internal node and unsequenced tips in the phylogenetic tree. The program output the inferred metagenome represented by KEGG Orthology for each FMC. Taking the PICRUST KO gene abundance inferences as inputs, the metabolic pathways were re-constructed using HUMAnN (v0.98) [34]. 

### Statistics

Nearest shrunken centroids classification was performed using pamr package in R (version 2.13.1). For the classification using both rectum and descending colon samples, 30 of the 451 genus-region variables with at least one nonzero component were selected at Δ = 1.459. For the classification using rectum samples only, 39 of the 226 genera with at least one nonzero component were selected at Δ = 1.030. The leave-one-out error rate was estimated by setting the number of cross-validation folds as 47 (which equals the number of subjects in the dataset). 

## Results

**Figure 1:**
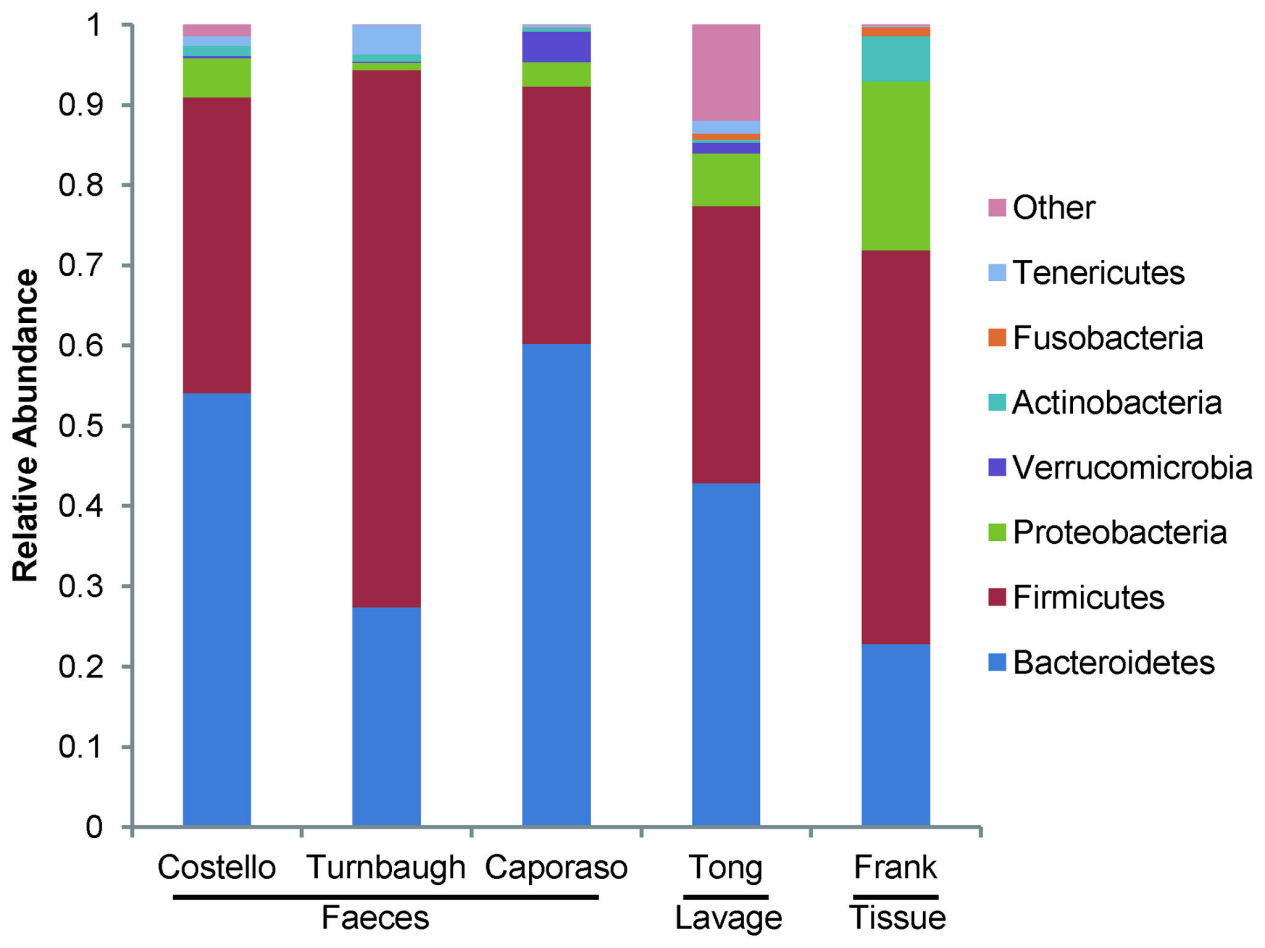
Phylum level microbial compositions of faeces, lavage and tissue samples. Biospeciemens from faeces (Costello [45], Turnbaugh [12] and Caporaso [11]), lavage samples (Tong) and tissue samples (Frank [21]) were compared. Only predominant phyla with relative abundances higher than 0.1% in Tong dataset were depicted in the bar graph, and the phyla with low abundances were grouped together. For Costello and Caporaso datasets, only the fractions of intestinal microbiota were shown here.

To study the host-microbial interaction at the mucosal luminal interface, 179 lavage samples were collected from different intestinal regions of 64 subjects; this cohort and these samples were previously used in a metaproteomic study of IBD [23] (Table S1). The microbiota from these samples were profiled by multiplex sequencing, and a total of 1,236,641 reads (6,909/sample on average) were generated after quality control. 10,208 species level OTUs were then generated by collapsing the reads at a 97% sequence similarity threshold. At the phylum level, the bacterial community from lavage sample mainly consisted of Bacteroidetes (44.29%), Firmicutes (35.48%), Proteobacteria (6.76%), Tenericutes (1.63%) and Verrucomicrobia (1.35%) (Figure 1). Other phyla were also detected at relatively low abundances (<1%) including Actinobacteria and Fusobacteria. We compared our dataset with other 16S sequence datasets generated from fecal or tissue samples after re-processing them using the same OTU picking and taxonomy assignment algorithms. Given the difference of sequencing platforms, primer sets and colon regions (Table S2), the Tong dataset was comparable with other intestinal microbial datasets in terms of microbial composition and phylotype richness. 

### Shifts of Microbial Composition in IBD Patients

**Figure 2:**
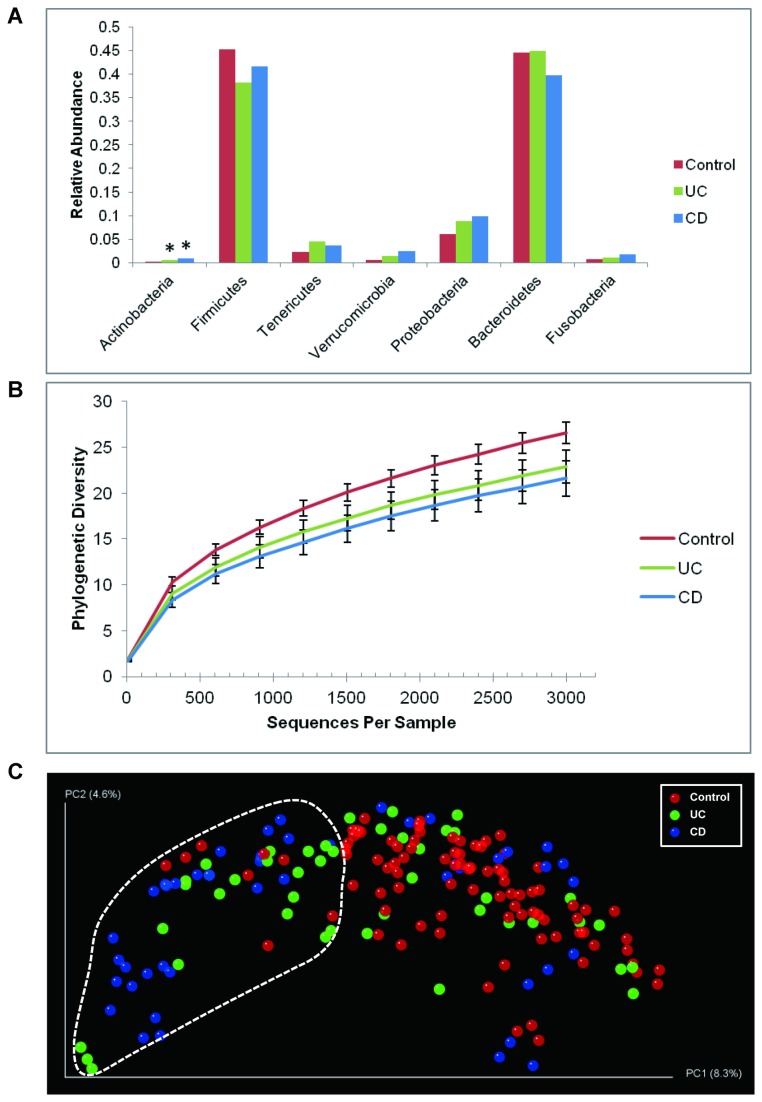
Shifts of mucosal microbial composition in IBD patients in remission. (**A**) The change of relative abundance between disease states at phylum level. *: *P* < 0.05 compared to control, ANOVA. (**B**) Phylogenetic diversity curves for the microbiota from lavage samples. Mean ± 95% CI was shown. (**C**) Communities clustered using PCoA of the unweighted UniFrac distance matrix. Each point corresponds to a sample colored by disease phenotype. The dotted line indicated the cluster of samples enriched for IBD subjects.

The IBD-associated dysbiosis of mucosal microbiota has been delineated in detail in several investigations [9,32,35]. Specifically, IBD patients have fewer Firmicutes and a concomitant increase in Proteobacteria, validated in several independent cohorts [36,37]. To determine whether previously reported alterations were also observed in our dataset, we compared the relative abundances of each phylum between disease states using analysis of variance (ANOVA). In contrast to controls, IBD patients harbored relatively more abundant Actinobacteria (FDR corrected *P* = 0.006 for UC, < 0.0001 for CD), accompanied with the depletion of Firmicutes (FDR corrected *P* = 0.056 for UC, 0.25 for CD) in these subjects (Figure 2A, Dataset S1 and S2). The increases of Proteobacteria (FDR corrected *P* = 0.254 for UC, 0.143 for CD) and Tenericutes (FDR corrected *P* = 0.115 for UC, 0.157 for CD) were also observed in IBD patients, although not statistically significant. Taken together, microbial composition represented by this study cohort, and captured by lavage sampling, reflected the changes of relative abundances of enteric microbiota in IBD subjects at phylum level observed in other datasets and sampling methods. 

The reduction in bacterial diversity in IBD patients is a consistent finding across studies [32,38,39], although it is still unknown whether this alteration is causative or a secondary effect of IBD. Compared with controls, the phylogenetic diversities of UC and CD subjects at 97% OTU level were significantly lower (Figure 2B), and the difference was more evident in CD (t-test, *P* = 0.0003) than that in UC subjects (t-test, *P* = 0.0056) at the depth of 3,000 reads per sample. This data indicates that the lower microbial diversity previously observed in patients with active IBD also persists in clinically quiescent phases of disease.

To evaluate the similarity between microbial communities in lavage samples from control and IBD subjects, the beta-diversity measured by unweighted distance matrix was calculated for each sample. The principal coordinate analysis (PCoA) plot showed that the samples clustered by diagnosis (Figure 2C). The IBD-associated dysbiosis of mucosal microbiota was reflected by the cluster of samples enriched for IBD, especially CD subjects. 55% of the IBD samples (49% of UC and 61% of CD) were in this cluster, whereas only 8% of the controls were in this IBD enriched clusters. The clustering was evident considering the heterogeneity of the pathogenesis of IBD [40], although control samples can be observed in the IBD enriched cluster and not all the IBD samples were grouped into this subset. 

A critical clinical question is whether we can develop quantitative microbial biomarkers to monitor differences in microbial composition associated with established IBD, because the absence of such biomarkers is currently a barrier to developing and assessing treatments targeting IBD-associated dybiosis. To develop such a tool, we determined whether the relative abundances of genera in the mucosal lavage samples could classify subjects by disease states, using a predictive model based on nearest shrunken centroids analysis [41]. Among the 64 subjects, we chose the 47 subjects where matched samples from both descending colon and rectum were available. Using 30 of the 451 genus-region variables, the optimal classification was achieved with the leave-one-out cross-validated error rate of 18/47 (38.3%) (Methods and Figure S1). We further assessed the effectiveness of disease discrimination based only on rectal samples, as these can be collected in a simple office procedure [42]. Strikingly, the error rate of classification, when using 39 genera from rectum samples only, was even lower (14/47, or 29.8%) (Figure S1). Considering that the IBD patients in our cohort were all in remission, this data demonstrated the potential of using microbial signatures as a disease classifier, but also suggested that higher precision than analysis of taxa independently is required for clinical utility. 

### Defining a microbial co-occurrence network at the intestinal mucosal surface

Extensive inter-species interactions are likely to operate among mucosal-associated microbiota residing in the complex and functionally diverse ecosystem of the intestines, either locally through formation of biofilms or through diffusion of nutrients and metabolites longitudinally along the intestine [43,44]. Such interactions can thus be potentially reflected by the co-occurrence and co-exclusion patterns inferred from abundance profiles of phylotypes [45]. Therefore, in addition to individual phylotypes, we must identify IBD-associated microbial community structures. To test the hypothesis that interactions among microbes increase our ability to classify samples according to clinical state, we constructed the microbial co-occurrence network using an approach specifically tailored for the 16S profiling data (Figure S2). The edge connecting each pair of nodes was the co-occurrence estimate inferred from the relative abundance profiles of genera using the sparse correlation measure SparCC [46], which ranged from -0.541 to 0.774, suggesting strong co-exclusion and co-occurrence relationships between phylotypes. As described in Methods, we transformed the SparCC correlation measure into a weighted network. 

### Identification of highly preserved functional microbial communities (FMCs)

To understand the topological structure of a network, one crucial step is to define modules, which are groups of highly connected nodes. In biological networks, modules can correspond to functional subunits such as protein complexes [47] or molecular pathways [48]. There is an extensive literature on clustering procedures, including simple k-means, partitioning around medoid, hierarchical clustering, message passing and model-based methods[49-52]. To determine if the genera in the microbial co-occurrence network can form network modules, we adapted weighted correlation network analysis (implemented in the WGCNA package) to construct microbial modules which can be interpreted as functional microbial communities (FMCs). WGCNA uses a measure of shared protein neighbors (based on the topological overlap measure) as input of hierarchical clustering. The height in the dendrogram is a measure of dissimilarity based on the topological overlap matrix; modules are defined as branches of a hierarchical cluster tree[29,31]. WGCNA is attractive in our study since it provides module preservation statistics that allowed us to assess the reproducibility of modules across different data sets; provides a measure of intramodular connectivity that can be used to define intramodular hub genera[53]; and, allows us to summarize each module by its module eigengenus. 

**Figure 3:**
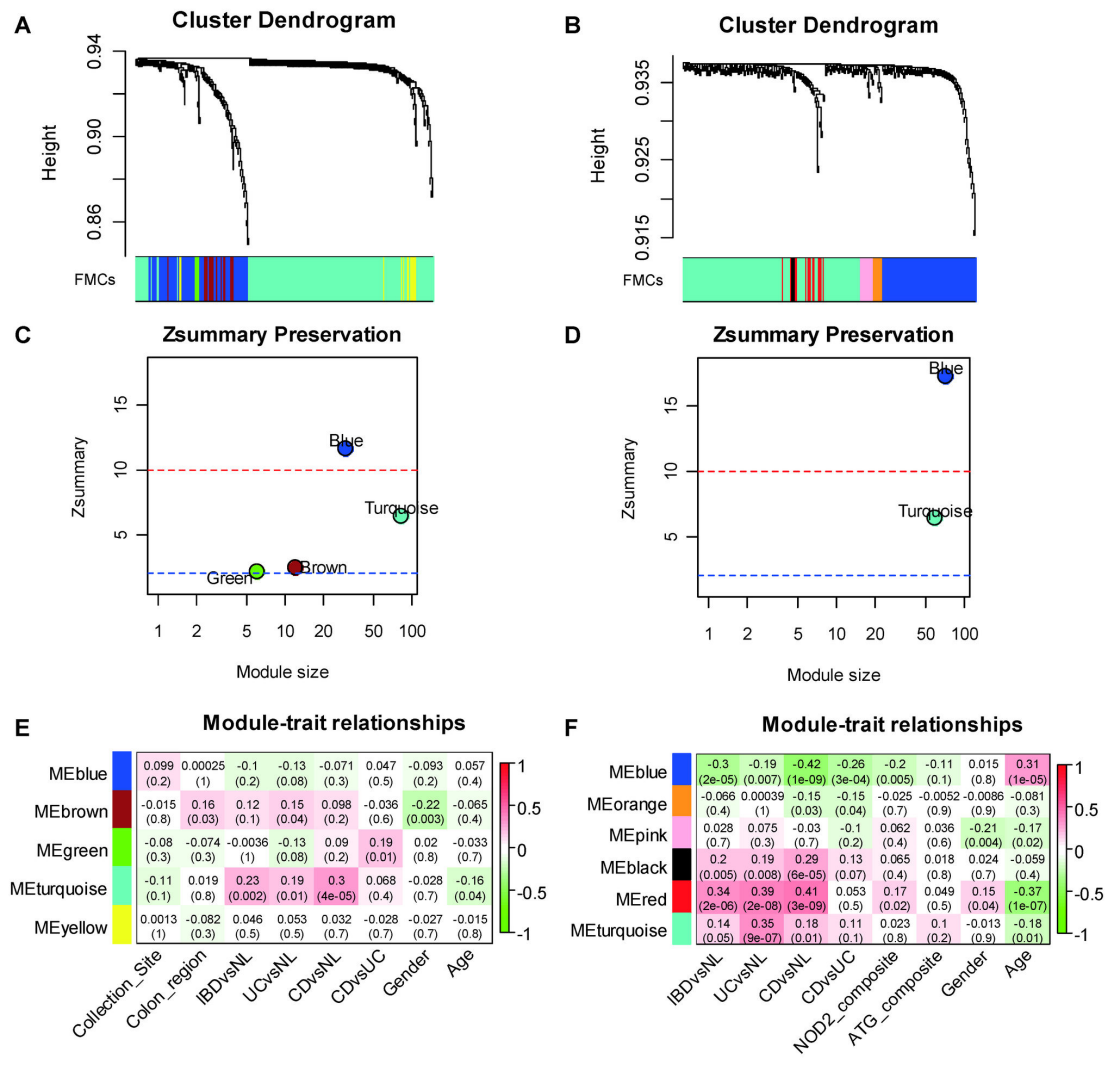
Identification of preserved functional microbial communities (FMCs) associated with disease phenotype across studies. Hierarchical clustering dendrograms of genera based on microbial co-occurrence network using the Tong dataset (**A**) and the Frank dataset (**B**) are shown. In the dendrograms, each color represents one FMC, and each branch represents one genus. The Z-summary statistic plots (y-axis) as a function of the module size are shown for the Tong dataset (**C**) and the Frank dataset (**D**). Each point represents a module labeled by color. The dashed blue and red lines indicate the thresholds Z = 2 and Z = 10, respectively. FMC-trait correlations and *P* values of the Tong dataset (**E**) and the Frank dataset (**F**). Each cell reports the correlation coefficient (and *P* value) derived from correlating FMC eigenvectors (rows) to traits (columns). The table is color-coded by correlation according to the color legend. Collection site: University of California Los Angeles or Cedars Sinai Medical Center; Colon region: 5 anatomical regions coded from 0 to 5, which are cecum, ascending colon, transverse colon, descending colon and rectum.

We first calculated the pair-wise topological overlap matrix after the soft-thresholding step to reduce the noise-level weak correlations (Methods). After grouping the nodes based on their topological overlaps using hierarchical clustering, we identified 5 functional microbial communities (Figure 3A), which consisted of 5 to 167 phylogenetically diverse genera (Table S3 and Dataset S3). Using the same method, analysis of the Frank dataset (263 genera) identified 6 microbial modules (Figure 3B), with a similar numerical range of genera per module (Table S3). The Tong and Frank datasets shared 129 genera. This reduced, common set of shared phylotypes yielded a similar module organization: 4 modules in the Tong dataset, and 2 modules in the Frank dataset (Figure S3). The reduced number of modules in the Frank dataset was probably due to the relatively low sequencing depth. The module memberships of the original and reduced datasets were highly concordant (Table S4, S5 and Dataset S3). 

To quantitatively evaluate the degree of module preservation, we carried out a Z-summary test (Methods). Alternative statistics are available to assess the quality and reproducibility of clusters among datasets [33,54-58]. An advantage of the Zsummary statistic is that it allows for significance thresholds: Z-summary <2 indicates no significant module preservation; 2<Z-summary<10 indicates moderate preservation; and, Z-summary>10 indicates strong preservation. Also, in previous work comparing WGCNA's module preservation statistics to a robust alternative method (the in-group proportion test of Kapp and Tibshirani [59]), both tests were highly correlated under a number of simulation conditions, and the Zsummary statistic had distinct advantages for studying the preservation of network modules[31,33,60]. 

As expected, all 4 modules of the shared 129 genera from the Tong dataset were highly preserved in the Frank dataset (Figure 3C), with the blue FMC demonstrating the strongest preservation. Conversely, all the modules in the Frank dataset were also well-preserved in the Tong dataset (Figure 3D). Given the methodological differences between Tong and Frank datasets, the co-occurrence pattern of these genera can still be observed at mucosal surface. We expected even more significant preservation when comparing datasets collected from same compartment and analyzed using same methodology. Indeed, when using another lavage sample dataset, referred hereafter as the mucosal luminal interface or MLI dataset, as the reference, 4 modules of the shared 233 genera from the Tong dataset were highly preserved in the MLI dataset (Table S6), with much higher Z-summary statistics (Figure S4). Thus, there were 2 core modules (turquoise and blue) in these datasets, despite the difference of sampling methods. These results indicated that the FMCs identified using our approach were not dataset specific, but robust and reproducible ecological structures commonly existing at the intestinal mucosal surface. 

### Identification of functional microbial communities (FMCs) associated with IBD

An optimal summary of the genus abundance profiles of a given FMC is the module eigengenus (defined in Methods). In the Tong dataset, we found that the turquoise FMC was significantly associated with Crohn’s disease state (*P* = 4 × 10^-5^, Pearson correlation) (Figure 3E). The blue FMC was negatively associated with IBD states, although not statistically significant. If the turquoise FMC was merely a group of individual CD-associated genera, it would include most of the 17 genera that were significantly enriched in CD samples (ANOVA, FDR corrected *P* < 0.05) (Dataset S4). However, only 7 of them were assigned to the turquoise FMC, indicating that FMCs also captured other intricate and underlying ecological relationships. Strikingly, classification of IBD status using the two core FMCs as quantitative microbial biomarkers achieved higher accuracy (17/47, or 36.2%) compared to using individual genera (Figure S1), indicating that the microbial modules allowed quantitative and reproducible microbial monitoring of the intestinal mucosa. Because the two core FMCs were highly preserved in both datasets, the same associations were also observed in the Frank dataset. The turquoise FMC was positively associated with IBD states, most significantly with UC (*P* = 9 × 10^-7^, Pearson correlation), whereas the blue FMC was negatively associated with CD (*P* = 1 × 10^-9^, Pearson correlation) (Figure 3F). The associations were stronger in the Frank dataset than those in the Tong dataset, possible because the samples were from patients with active disease. Consistent with previous observation [9], the blue FMC was also negatively associated with the *NOD2* risk allele in the Frank dataset, supporting the hypothesis that the CD-associated dysbiosis was driven by the *NOD2* risk allele [6,61]. 

After defining modules, we sought to analyze them by intuitive topological concepts such as intramodular connectivity, to better describe the network structure. Therefore, we determined the kME value (intramodular connectivity based on the module eigengenus) to define the correlation between each genus and the respective module eigengenus. Because nodes with high connectivity, i.e. the hubs, are centrally located within the module, they may be functionally essential as keystone species in the context of biological networks [62] and during the assemblage of a disease associated FMC. Indeed, in the turquoise FMC, the intramodular connectivities of the genera enriched in CD samples were significantly higher than those of the other members (t-test, *P* < 0.001) (Figure S5). Potential pathobiont genera such as *Enterococcus* [63] and *Escherichia* (including adherent-invasive *Escherichia coli* [64]) can also be observed among the hub genera of CD-associated turquoise FMC. In the blue module, one of the intramodular hub genera was *Faecalibacterium*, a genus including the anti-inflammatory commensal bacterium *Faecalibacterium prausnitzii*, that is negatively associated with Crohn’s disease [35,65,66]. Accordingly, the relative abundance of *Faecalibacterium* decreased by 2-fold in Crohn’s disease samples (ANOVA, FDR corrected *P* = 0.006, Dataset S2). Other short-chain fatty acid (SCFA) producing bacteria including *Eubacterium*, *Roseburia*, *Faecalibacterium* and *Coprococcus* were also observed in the blue FMC [67-69]. Taken together, these data demonstrated the functional importance of the FMCs associated with CD. 

### Metabolic inference and reconstruction of functional microbial communities

**Figure 4:**
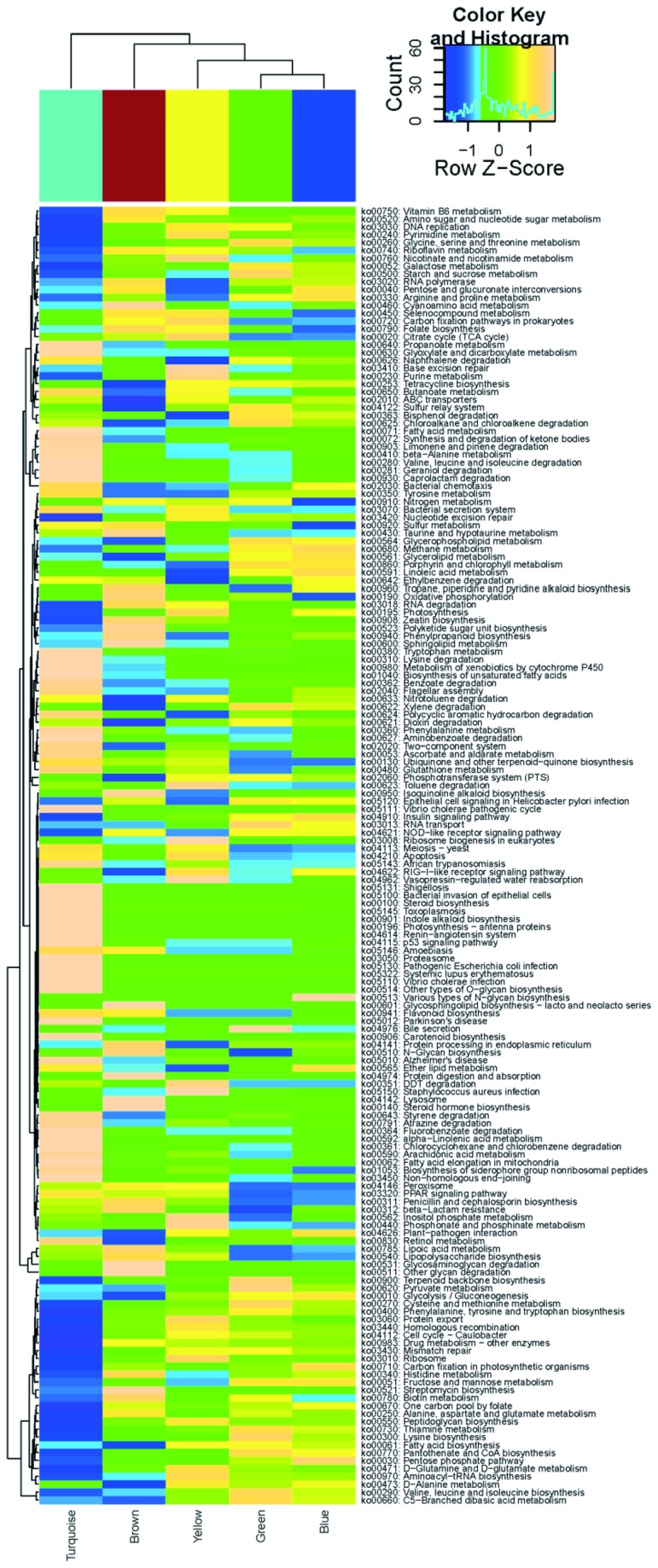
Variations of KEGG metabolic pathways in the functional microbial communities. The heatmap shows the functional profiles of FMCs (columns) based on the relative abundance of KEGG metabolic pathways (rows) after *z* score transformation. The color bar on top shows module membership. The dendrograms show the hierarchical clustering of columns and rows respectively using Euclidean distance.

The disease associations of the well preserved FMCs suggest that these co-occurred microbial communities represent distinct functional units at the mucosal surface. To profile the metabolic capabilities of FMCs, the approximate gene contents of the detected phylotypes in each FMCs were inferred using the 1,119 KEGG reference genomes. After aggregating the individual inferred genomes according to module membership, the relative abundances of metabolic pathways in each FMC were re-constructed. The functional profiles of FMCs were significantly variable (Figure 4). The representation of the functional groups that were likely essential for life in the gut was highly consistent across FMCs including those for carbohydrate and amino-acid metabolism (for example glycolysis/gluconeogenesis (KO00010), pyruvate metabolism (KO00620) and glycine, serine and threonine metabolism (KO00260)). In contrast, several virulent pathways including bacterial invasion of epithelial cells (KO05100) and pathogenic *Escherichia coli* infection (KO05130) were only present in the IBD-associated turquoise FMC. Variably represented pathways included glycan degradation (KO00511) and glycosaminoglycan degradation (KO00531), which were over-represented in the UC-associated brown FMC; and, glutathione metabolism (KO00480), which was enriched in turquoise FMC (Dataset S5). With respect to the former, murine defects in mucosal barrier function due to depletion of intestinal *O*-glycans causes spontaneous colitis [70]. Regarding the latter, an increase in glutathione metabolism is a feature of intestinal microbiome in inflammatory bowel disease [71]. These observations, combined with the disease association, indicated that the imputed virulent metabolic functions carried out by the disease associated FMCs contributed to the pathogenic and chronic inflammatory state of intestinal mucosal surface. 

## Discussion

We have developed a novel strategy using an ecologic mucosal microbial framework, minimally invasive mucosal sampling, short-read Illumina sequencing, network analysis, and imputed metagenomics. This strategy uncovered 5 microbial modules detectable in the mucosa of all individuals, and reproducible in an independent mucosal resection dataset (Figure 3). The quantitative levels of two modules were significantly associated with disease states (Figure 3E). More than 70% of the subjects can be correctly classified as control or IBD patients using genera from rectal sampling alone (Figure S1), which is a minimally invasive procedure compared to endoscopic biopsy, and thus notable for clinical translation. Imputed metagenome analysis indicated the functional importance of the disease associated modules reflected by the enrichment of virulent and pathogenic pathways (Figure 4). Thus, these modules appear to define novel microbial communities within the intestinal microbial ecology, some of which are commonly and stably modified by the IBD disease state, and may be of particular relevance for microbial pathogenesis and intervention.

Mucosal sampling and module analysis provided robust differentiation at the individual patient level that has not been achieved previously by analysis of the fecal compartment and conventional analyses (individual phylotypes levels, community alpha-diversity, or principal component analysis) [36,37]. How might the design features of the study have contributed to this outcome? One distinction was the first use of mucosal lavage for depth microbial analysis. Lavage samples microbiota embedded in the superficial mucin, but may also include luminal fecal residue remaining after intestinal preparation. Mucosa-associated microbial composition varies across segments of the intestine, and distinct as well from the fecal compartment [24,72]. Due to the predominant inter-individual signal, it is uncertain whether the lavage compartment yields a distinct microbial composition from mucosal or fecal sampling. 

Nonetheless, compared to fecal samples, mucosal lavage is from a defined (~1 cm^2^) area of mucosal surface [23], and therefore captures a local microbial community more homogeneous for local metabolic exchange and interaction in the microenvironment. Indeed, since the local habitat modifies the functional state of the microbial community, lavage samples can be analytically extended to define microbial state (by transcriptional and metagenomic analysis of the bacterial pellet) and the habitat (by biochemical analysis of supernatant proteins and metabolites). We have recently reported high yields of soluble fraction proteins and metabolites by lavage sampling, and have uncovered robust, disease-specific biochemical features of the mucosal surface [23]. Lavage sampling, by permitting microbial and biochemical analysis from the same mucosal site, could be extended to integrated multi-omic analysis to functionally characterize the intestinal microbial ecosystem. And, owing to its noninvasiveness, lavage sampling in contrast to biopsy or surgery permits longitudinal sampling which is an important barrier to monitoring the mucosal microbiota and its dynamic temporal state [11,24].

A second distinction of this study was the use of WGCNA module analysis, built on co-occurrent or co-exclusive microbiota, to uncover functional microbial communities within the intestinal mucosal compartment. Prior studies of the fecal compartment have not reported such microbial modules between individuals, and early findings of recurrent communities dominating the entire fecal microbiome [73] have not been consistently observed [13,74-76]. Recently, Faust et al. analyzed the HMP dataset to define co-occurrence and co-exclusion microbial interaction networks within and between 18 body sites [22]. Among these, only the vaginal compartment revealed a robust modular community structure. In the fecal compartment, this study reported 67 interactions (2.2% of total phylotypes), but limited modular features (for example, Bacteroides and Prevotellaceae co-excluded), presumably reflecting metabolic specialization and niche competition of these family members based on diet and other factors [13,73-75]. It should also be noted that the HMP dataset is from healthy subjects only, and from a much larger population. Such characteristics therefore potentially explain a decrease of the modular aspect in the stool samples. 

The present study uncovered similar networks of co-occurrent and co-exclusive microbiota, confirming shared features detected in the fecal and mucosal lavage compartments. In addition, extending the analysis to WGCNA uncovered 5 reproducible microbial modules, each comprised of distinct but phylogenetically mixed group of organisms, and a blend of positive and negative microbial interactions. We have termed them functional microbial communities (FMCs), with the speculation that they reflect a physically localized and biologically integrated microbial network. Since each module is defined by both positively and negatively microbial interactions, we speculate that they will be defined by a distinctive ensemble of biologic factors, such as host microenvironment and microbial gardening, microbial cross-feeding and competition, and microbial small molecule and environmental modification [77-80]. 

Therapeutic intervention targeting microbial dysbiosis in inflammatory bowel disease is an important prospect for changing the natural history for patients with inflammatory bowel disease. However, the heterogeneity and temporal variation of microbial composition requires new concepts to define the target of microbial intervention, and analytic tools to accurately sub-stratify and monitor individual patients. In our study, all 5 of the FMCs identified were present in all the subjects, but with different overall abundances that varied with disease states. In the CD-associated turquoise FMC, the difference of intramodular connectivity suggested that the pathogenic microbes were more likely to be the core members of the microbiota, rather than opportunistic pathogens. Direct evidence for the physical localization of such ecological structures could be validated using methods such as fluorescence *in* *situ* hybridization, whereas functional features of these communities would require comprehensive metagenomic or biochemical analysis. In this respect, a recent metaproteomic study of the mucosa surface detected a physical microgeographic mosaic of proteins, which might represent a biochemical counterpart to the microbial modules (Li X et al., submitted). 

Meta-analysis of genome-wide association studies (GWAS) has increased the number of confirmed IBD susceptibility loci to 99 [81,82], indicating that IBD is biologically heterogeneous. This heterogeneity may also extend to the microbial level, as a subset of IBD samples in our dataset clustered with control samples in the PCoA plot, and 4 IBD subjects were incorrectly categorized as controls based on their rectum microbial compositions in the shrunken centroids analysis. Host genetic factors are contributors shaping individual microbiome diversity in mammals [7,9,83]. The low disease penetrance in individuals carrying disease risk loci favors the hypothesis that several genetic and environmental factors interact to cause IBD [84]. These results presented here, combined with host genetic information, serve as an important step towards understanding the factors that govern the assemblages of gut microbiota associated with IBD. The understanding of the ecological interactions that govern the assemblage of FMCs will help us design interventions that counteract the environmental or genetic factors that cause perturbation of FMCs into unhealthy states, and perhaps shift our search of the causes of IBD away from detection of specific pathogens and towards dysregulation of microbes that are harmless or beneficial in other contexts but are dangerous weeds in the context of IBD. 

## Supporting Information

Table S1 **Demographic information of Tong dataset.** Note: on average, 3 samples of different intestinal regions were collected from each subject. UC, ulcerative colitis; CD, Crohn’s disease; CE, cecum; AS, ascending colon; TR, transverse colon; DE, descending colon; RE, rectum.
(PDF)

Table S2 **Comparison of published intestinal microbiota datasets.** The factors that may affect the microbial compositions, including technical parameters of sequencing pipeline (platform, variable region, primer set) and sample type, are listed.
(PDF)

Table S3 **Sizes of FMCs in the Tong Total, Tong Overlap, Frank Total and Frank Overlap datasets.**
(PDF)

Table S4 **Module membership comparison between FMCs from the Tong dataset and those from the shared phylotypes in Tong dataset.**
(PDF)

Table S5 **Module membership comparison between FMCs from the Frank dataset and those from the shared phylotypes in Frank dataset.**
(PDF)

Table S6 **Module membership comparison between FMCs from the Tong dataset and those from the Tong-MLI shared dataset.**
(PDF)

Figure S1 **Classification of control and IBD subjects using nearest shrunken centroids analyses of the relative abundances of bacterial genera and FMCs from lavage samples.** Only subjects (n = 47) that had matched samples from both descending colon and rectum regions were included in the analysis. Control and IBD samples with leave-one-out cross-validated probabilities higher than 50% were considered correctly classified. Diamond, classification using 30 genus-region variables (error = 18/47, or 38.3%); Square: classification using 39 rectum genera variables (error = 14/47, or 29.8%); Triangle, classification using 4 FMC-region variables (error = 17/47, or 36.2%).
(PDF)

Figure S2 **Overview of the methodology for inferring microbial co-occurrence network and identifying functional microbial communities.**
(PDF)

Figure S3 **Identification of functional microbial communities in co-occurrence network of 129 shared genera.** Hierarchical clustering dendrograms of genera based on microbial co-occurrence network using the Tong dataset (A) and the Frank dataset (B) are shown. In the dendrograms, each color represents one FMC, and each branch represents one genus.
(PDF)

Figure S4 **Preservation of FMCs in Tong dataset using MLI as reference.** The Z-summary statistic plots (y-axis) as a function of the module size are shown for the Tong dataset. Each point represents a module labeled by color. The dashed red lines indicate the thresholds Z = 10.
(PDF)

Figure S5 **Intramodular connectivity of CD enriched genera and other members in turquoise FMC of Tong datset.** Mean ± standard error is shown.
(PDF)

Dataset S1 **OTU significance results between UC and control subjects.**
(XLSX)

Dataset S2 **OTU significance results between CD and control subjects.**
(XLSX)

Dataset S3 **Lists of module memberships of Tong, Frank, and shared datasets.**
(XLSX)

Dataset S4 **Intramodular connectivity of genera in each FMC from Tong dataset.**
(XLSX)

Dataset S5 **Relative abundances of metabolic pathways in each FMC from Tong dataset.**
(XLSX)
