# Supplementary material for: A Modular Organization of the Human Intestinal Mucosal Microbiota and Its Association with Inflammatory Bowel Disease
Source: PLoS One. 2013 Nov 19;8(11):e80702. doi: 10.1371/journal.pone.0080702 (PMC3834335; doi:10.1371/journal.pone.0080702)

Table S1. Demographic information of Tong dataset.

Metadata of Tong Dataset				
		Control	UC	CD
Subject (64)		32	16	16
Gender	Male	20	9	11
	Female	12	7	5
Age (Average ± SD)		60 ± 12	36 ± 12	41 ± 12
Sample (179)		90	43	46
Anatomical region	CE (1)	1	0	0
	AS (47)	25	15	7
	TR (22)	4	8	10
	DE (57)	31	12	14
	RE (52)	29	8	15

Table S4. Module membership comparison between FMCs from the Tong Total and Tong Overlap dataset.

		Tong Total				
		Blue (40)	Brown (12)	Turquoise (60)	Green (5)	Yellow (12)
Tong Overlap	Blue (30)	**29**	1			
	Brown (12)	1	**11**			
	Turquoise (81)	9		**60**		12
	Green (6)	1			**5**	

Table S5. Module membership comparison between FMCs from the Frank Total and Frank Overlap dataset.

		Frank Total					
		Turquoise (52)	Blue (55)	Red (3)	Orange (7)	Black (1)	Pink (11)
Frank Overlap	Turquoise (59)	**27**	11	3	6	1	11
	Blue (70)	25	**44**		1		

Table S6. Module membership comparison between FMCs from the Tong dataset and those from the Tong-MLI shared dataset.

		Tong Total				
		Blue (62)	Yellow (14)	Turquoise (137)	Green (5)	Brown (15)
Tong-MLI Overlap	Blue (49)	**35**				14
	Yellow (18)	1	**14**	3		
	Turquoise (161)	26		**134**		1
	Green (5)				**5**	

Figure S1

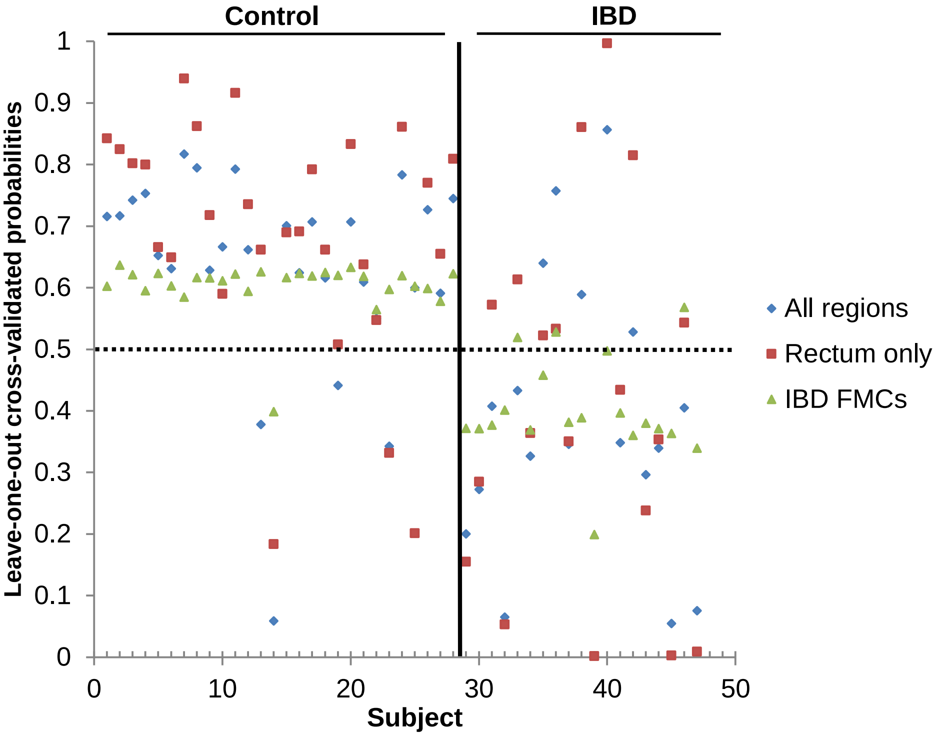

Figure S2

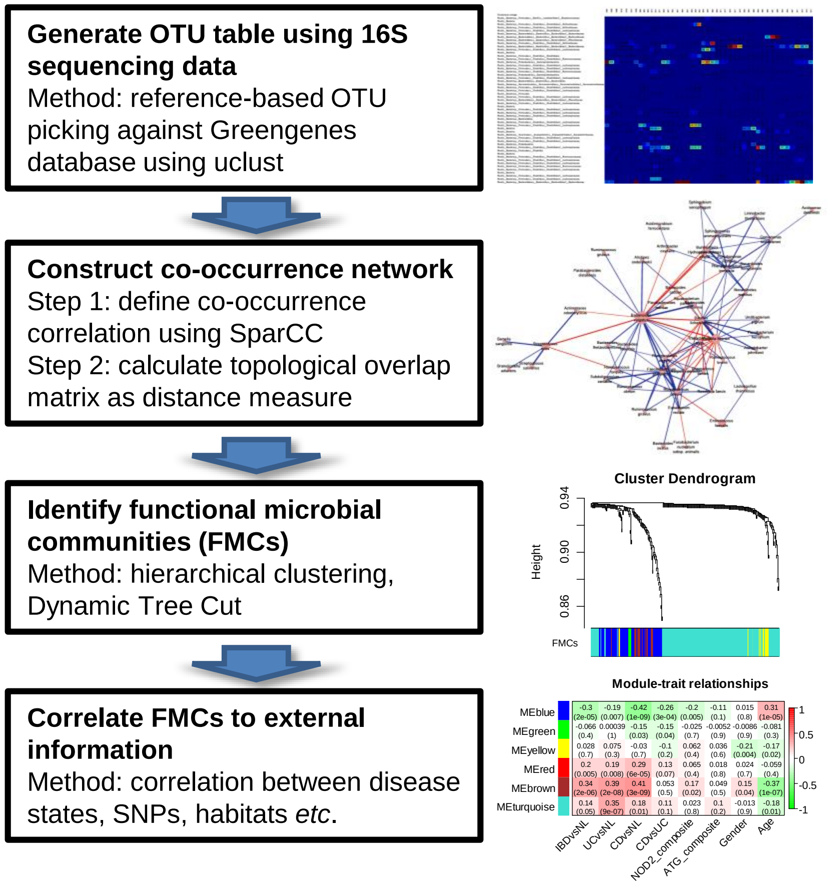

Figure S3

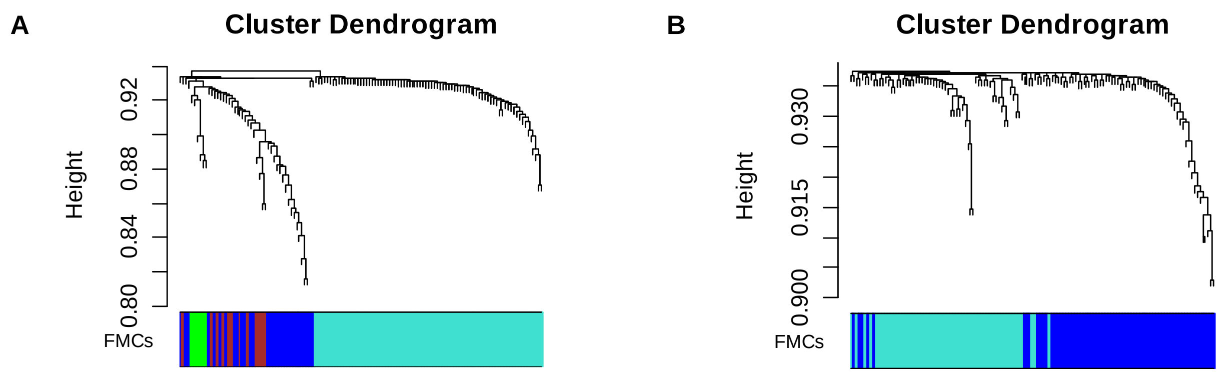

Figure S4

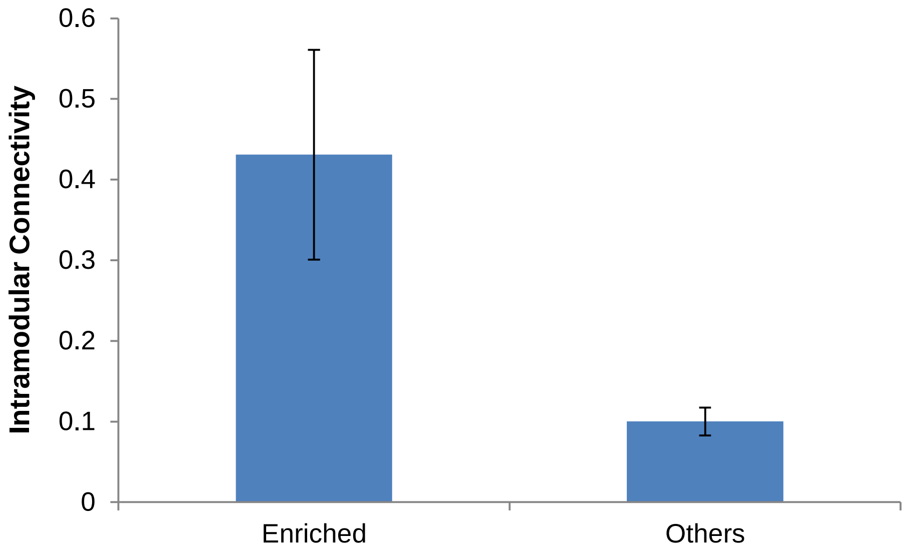

Figure S5

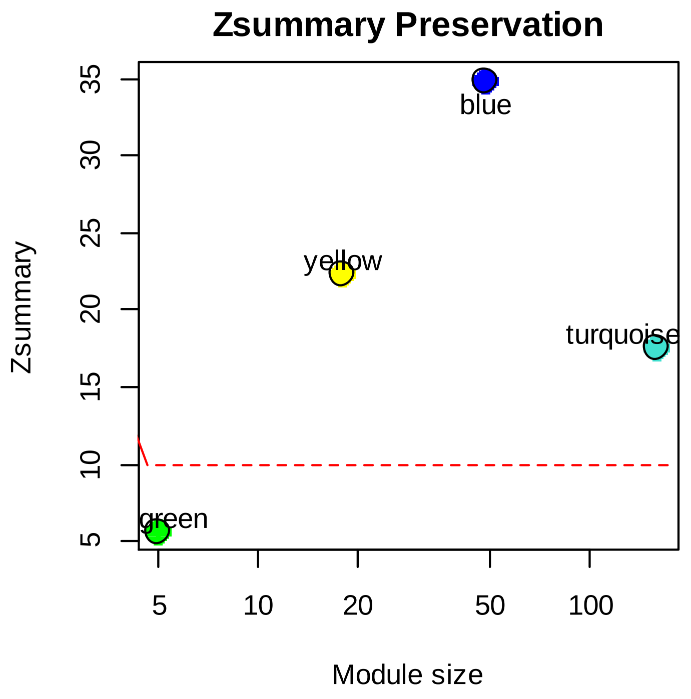

Supplement: Table S1 — Demographic information of Tong dataset. Note: on average, 3 samples of different intestinal regions were collected from each subject. UC, ulcerative colitis; CD, Crohn’s disease; CE, cecum; AS, ascending colon; TR, transverse colon; DE, descending colon; RE, rectum. (PDF) [file pone.0080702.s001.pdf]
