# Supplementary material for: A Modular Organization of the Human Intestinal Mucosal Microbiota and Its Association with Inflammatory Bowel Disease
Source: PLoS One. 2013 Nov 19;8(11):e80702. doi: 10.1371/journal.pone.0080702 (PMC3834335; doi:10.1371/journal.pone.0080702)
Supplement: Table S2 — Comparison of published intestinal microbiota datasets. The factors that may affect the microbial compositions, including technical parameters of sequencing pipeline (platform, variable region, primer set) and sample type, are listed. (PDF) [file pone.0080702.s002.pdf]

Table S2. Comparison of published intestinal microbiota datasets.

Dataset	Platform	Variable region	Primer set	Sample type	Subject number	Sample number	Reference
Tong	Illumina HiSeq 2000	V4	515F/806R	Lavage	64	179	
Frank	Sanger	V1-V4	8F/805R	Tissue	124	190	(24)
Costello	454	V2	27F/338R	Fecal	45	45	(65)
Turnbaugh	454	V2	27F/338R	Fecal	154	282	(14)
Caporaso	Illumina GAIIx	V4	515F/806R	Fecal	2	466	(11)
MLI	Illumina HiSeq 2000	V4	515F/806R	Lavage	148	285	
