# Supplementary material for: A Modular Organization of the Human Intestinal Mucosal Microbiota and Its Association with Inflammatory Bowel Disease
Source: PLoS One. 2013 Nov 19;8(11):e80702. doi: 10.1371/journal.pone.0080702 (PMC3834335; doi:10.1371/journal.pone.0080702)
Supplement: Table S3 — Sizes of FMCs in the Tong Total, Tong Overlap, Frank Total and Frank Overlap datasets. (PDF) [file pone.0080702.s003.pdf]

Table S3. Sizes of FMCs in the Tong Total, Tong Overlap, Frank Total and Frank Overlap datasets.

FMCs	Turquoise	Blue	Brown	Yellow	Green	Red	Pink	Orange	Black
Tong Total	167	62	15	14	5				
Tong Overlap	81	30	12		6				
Frank Total	142	84				14	12	8	3
Frank Overlap	59	70							
