# Supplementary material for: A Modular Organization of the Human Intestinal Mucosal Microbiota and Its Association with Inflammatory Bowel Disease
Source: PLoS One. 2013 Nov 19;8(11):e80702. doi: 10.1371/journal.pone.0080702 (PMC3834335; doi:10.1371/journal.pone.0080702)
Supplement: Table S4 — Module membership comparison between FMCs from the Tong dataset and those from the shared phylotypes in Tong dataset. (PDF) [file pone.0080702.s004.pdf]

Table S4. Module membership comparison between FMCs from the Tong Total and Tong Overlap dataset.

| | | Tong Total | | | | |
|---|---|---|---|---|---|---|
| | | Blue (40) | Brown (12) | Turquoise (60) | Green (5) | Yellow (12) |
| Tong Overlap | Blue (30) | **29** | 1 | | | |
| | Brown (12) | 1 | **11** | | | |
| | Turquoise (81) | 9 | | **60** | | 12 |
| | Green (6) | 1 | | | **5** | |
