# Supplementary material for: A Modular Organization of the Human Intestinal Mucosal Microbiota and Its Association with Inflammatory Bowel Disease
Source: PLoS One. 2013 Nov 19;8(11):e80702. doi: 10.1371/journal.pone.0080702 (PMC3834335; doi:10.1371/journal.pone.0080702)
Supplement: Table S5 — Module membership comparison between FMCs from the Frank dataset and those from the shared phylotypes in Frank dataset. (PDF) [file pone.0080702.s005.pdf]

Table S5. Module membership comparison between FMCs from the Frank Total and Frank Overlap dataset.

		Frank Total					
		Turquoise (52)	Blue (55)	Red (3)	Orange (7)	Black (1)	Pink (11)
Frank Overlap	Turquoise (59)	**27**	11	3	6	1	11
	Blue (70)	25	**44**		1		
