# Supplementary material for: A Modular Organization of the Human Intestinal Mucosal Microbiota and Its Association with Inflammatory Bowel Disease
Source: PLoS One. 2013 Nov 19;8(11):e80702. doi: 10.1371/journal.pone.0080702 (PMC3834335; doi:10.1371/journal.pone.0080702)
Supplement: Table S6 — Module membership comparison between FMCs from the Tong dataset and those from the Tong-MLI shared dataset. (PDF) [file pone.0080702.s006.pdf]

Table S6. Module membership comparison between FMCs from the Tong dataset and those from the Tong-MLI shared dataset.

		Tong Total				
		Blue (62)	Yellow (14)	Turquoise (137)	Green (5)	Brown (15)
Tong-MLI Overlap	Blue (49)	**35**				14
	Yellow (18)	1	**14**	3		
	Turquoise (161)	26		**134**		1
	Green (5)				**5**	
