# Supplementary figures and images for: A Modular Organization of the Human Intestinal Mucosal Microbiota and Its Association with Inflammatory Bowel Disease
Source: PLoS One. 2013 Nov 19;8(11):e80702. doi: 10.1371/journal.pone.0080702 (PMC3834335; doi:10.1371/journal.pone.0080702)

Figure S1

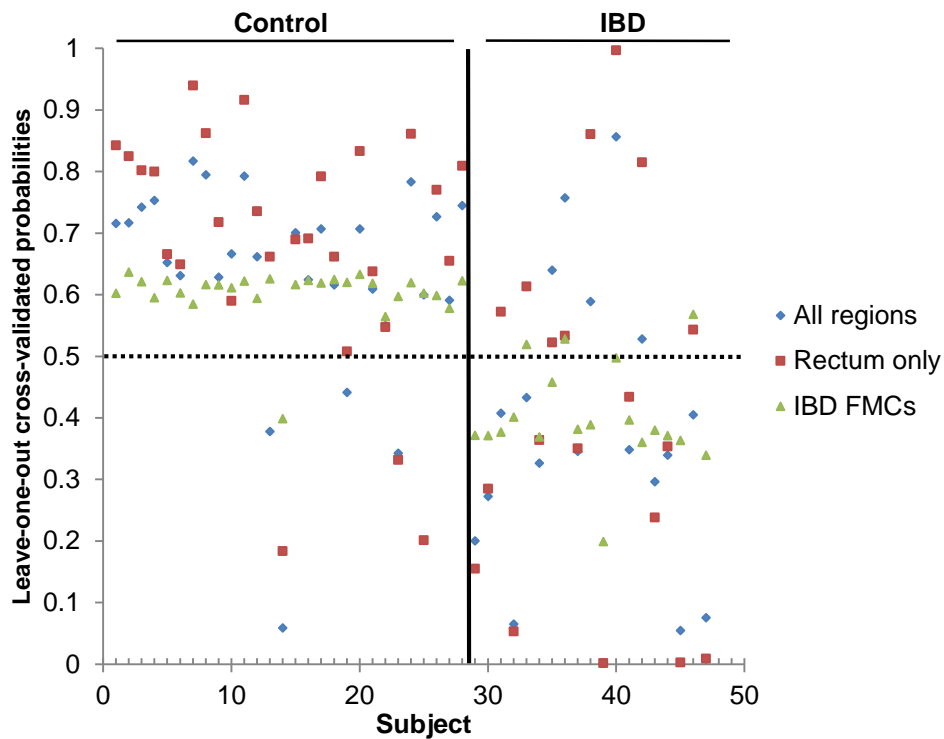

Supplement: Figure S1 — Classification of control and IBD subjects using nearest shrunken centroids analyses of the relative abundances of bacterial genera and FMCs from lavage samples. Only subjects (n = 47) that had matched samples from both descending colon and rectum regions were included in the analysis. Control and IBD samples with leave-one-out cross-validated probabilities higher than 50% were considered correctly classified. Diamond, classification using 30 genus-region variables (error = 18/47, or 38.3%); Square: classification using 39 rectum genera variables (error = 14/47, or 29.8%); Triangle, classification using 4 FMC-region variables (error = 17/47, or 36.2%). (PDF) [file pone.0080702.s007.pdf]

Figure S3

**A**

**Cluster Dendrogram**

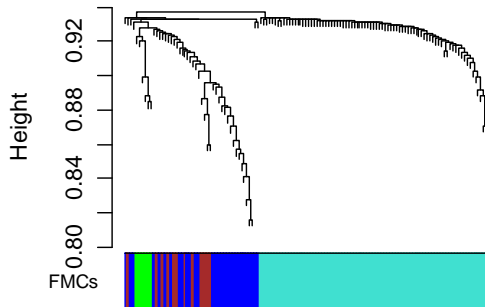

**B**

**Cluster Dendrogram**

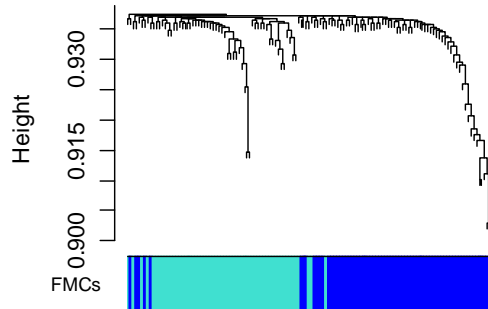

Supplement: Figure S3 — Identification of functional microbial communities in co-occurrence network of 129 shared genera. Hierarchical clustering dendrograms of genera based on microbial co-occurrence network using the Tong dataset (A) and the Frank dataset (B) are shown. In the dendrograms, each color represents one FMC, and each branch represents one genus. (PDF) [file pone.0080702.s009.pdf]

Figure S4

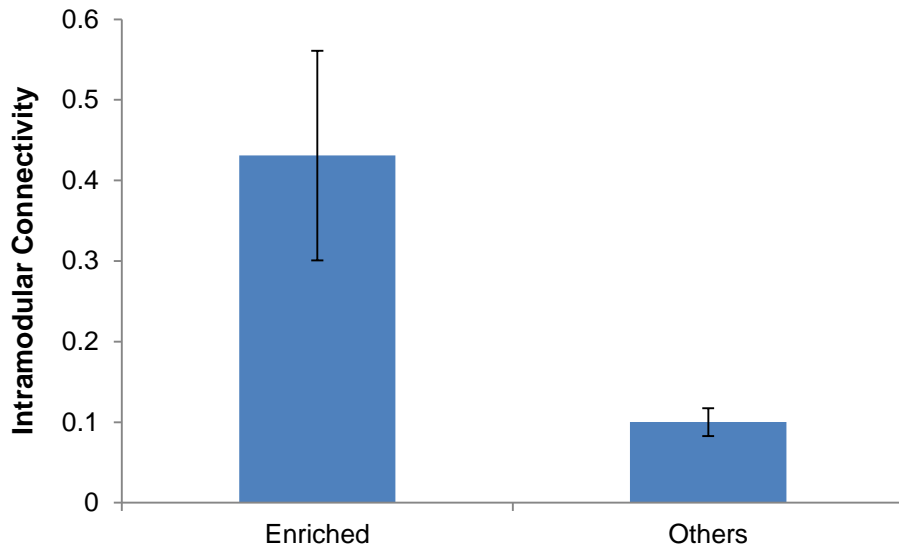

Supplement: Figure S4 — Preservation of FMCs in Tong dataset using MLI as reference. The Z-summary statistic plots (y-axis) as a function of the module size are shown for the Tong dataset. Each point represents a module labeled by color. The dashed red lines indicate the thresholds Z = 10. (PDF) [file pone.0080702.s010.pdf]

Figure S5

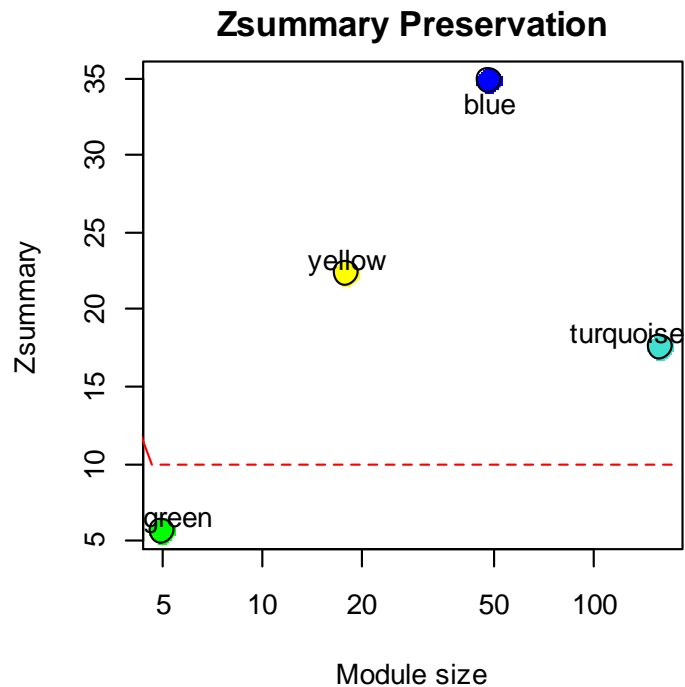

Supplement: Figure S5 — Intramodular connectivity of CD enriched genera and other members in turquoise FMC of Tong datset. Mean ± standard error is shown. (PDF) [file pone.0080702.s011.pdf]
