# Supplementary material for: A Modular Organization of the Human Intestinal Mucosal Microbiota and Its Association with Inflammatory Bowel Disease
Source: PLoS One. 2013 Nov 19;8(11):e80702. doi: 10.1371/journal.pone.0080702 (PMC3834335; doi:10.1371/journal.pone.0080702)

Figure S2

Generate OTU table using 16S sequencing data
Method: reference-based OTU picking against Greengenes database using uclust

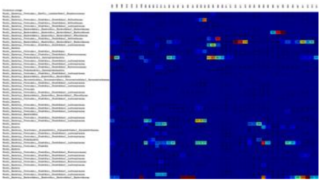

Construct co-occurrence network
Step 1: define co-occurrence correlation using SparCC
Step 2: calculate topological overlap matrix as distance measure

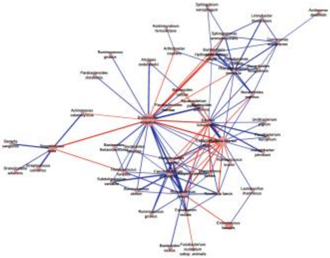

Identify functional microbial communities (FMCs)
Method: hierarchical clustering, Dynamic Tree Cut

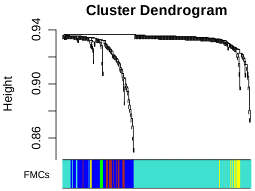

Correlate FMCs to external information
Method: correlation between disease states, SNPs, habitats *etc*.

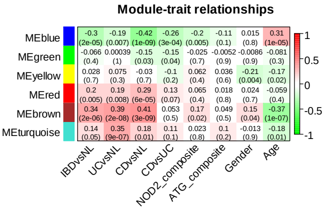

Supplement: Figure S2 — Overview of the methodology for inferring microbial co-occurrence network and identifying functional microbial communities. (PDF) [file pone.0080702.s008.pdf]
